# Changes in infant respiratory pathogens pre-, during, and post-COVID-19 non-pharmacological interventions in Beijing

**DOI:** 10.1186/s13052-025-01848-5

**Published:** 2025-01-22

**Authors:** Tongying Han, Yajuan Wang, Di Zhang, Ying Li, Li Zhang, Jin Yan, Chi Li, Shengnan Yang, Litao Guo, Huijuan Yan

**Affiliations:** Department of Neonatology, Children’s Hospital, Capital Institute of Pediatrics, Beijing, 100020 China

**Keywords:** Non-pharmacological interventions, Infants, Respiratory infections, Pathogeny

## Abstract

**Background:**

To explore the effect of non-pharmacological interventions (NPIs) on respiratory pathogen profiles among hospitalized infants aged 0–3 months in Beijing during the coronavirus disease 2019 (COVID-19) pandemic.

**Methods:**

Respiratory specimens were collected from 1,184 infants aged 0–3 months who were hospitalized for acute respiratory infection at the Children’s Hospital affiliated with the Capital Institute of Pediatrics from January 2018 to December 2023. The data were divided into three groups—the pre-epidemic (January 2018 to December 2019), epidemic prevention and control (January 2020 to December 2022), and post-epidemic (January 2023 to December 2023) groups—based on the outbreak of COVID-19 and the implementation and termination of NPIs. The specimens were tested for 14 respiratory pathogens, including influenza virus A (Flu A), influenza virus B, respiratory syncytial virus, parainfluenza virus (PIV), adenovirus (ADV), human metapneumovirus (HMPV), human bocavirus, human rhinovirus (HRV), coronavirus, *Chlamydia trachomatis*, *Chlamydia pneumoniae* (C.pn), *Mycoplasma pneumoniae*, *Bordetella pertussis*, and severe acute respiratory syndrome coronavirus 2 (SARS-CoV-2).

**Results:**

A total of 1,184 infants, including 649 males and 535 females, with acute respiratory infections were admitted. The positive detection rate for respiratory pathogens was 51.77% (*n* = 613). In 2023, the proportion of infants with respiratory infections after the epidemic was 19.4% (319/1646), the positive detection rate of respiratory pathogens was 68.3% (218/319), and the mixed infection detection rate of respiratory pathogens was 16.1% (35/218). Prior to the epidemic, these rates were 11.9% (431/3611), 37.1% (160/431), and 5.0% (8/160), respectively. During the epidemic prevention and control period, these rates significantly increased to 12.4% (434/3486), 54.1% (235/434), and 11.1% (26/235) (*P* < 0.05), respectively. Post-epidemic, the proportion of newborns testing positive for respiratory pathogens decreased, while the number of infants aged 29–90 days significantly increased. The proportion of admission weight and contact history with respiratory patients increased significantly compared to before and during the epidemic, with statistical significance (*P* < 0.05). After the epidemic, a total of 13 respiratory pathogens were detected throughout the year. There were statistically significant differences in the detection rates of Flu A, PIV, SARS-CoV-2, HRV, HMPV, ADV, and C.pn before, during, and after implementation of the NPIs during the COVID-19 epidemic (*P* < 0.05). Post-epidemic, the detection rates of Flu A, PIV, and SARS-CoV-2 were significantly higher than those before and during the epidemic (*P* < 0.017). The detection rates of HRV, HMPV, and ADV significantly increased after the epidemic compared to those before the epidemic (*P* < 0.017). Before the epidemic, the positivity rate of respiratory pathogens was high in the first and fourth quarters. After the termination of NPIs, the positive detection rate decreased in the first quarter but increased in the second, third, and fourth quarters, with a statistically significant difference (*P* < 0.05).

**Conclusion:**

The implementation and lifting of COVID-19 NPIs have caused significant changes in the detection and seasonal distribution of respiratory pathogens in infants aged 0–3 months in Beijing. NPIs temporarily reduced the detection rate of respiratory pathogens in infants during the prevalence of COVID-19. Understanding the prevalence of respiratory pathogens before and after the epidemic is particularly important for the prevention and control of respiratory diseases in infants.

## Introduction

Acute respiratory infections are prevalent among infants, attracting significant global attention owing to their incidence and associated mortality [1–3]. After the outbreak of coronavirus disease 2019 (COVID-19) in late 2019, countries implemented non-pharmacological interventions (NPIs), such as school closures, social distancing, mask-wearing, improved hand hygiene, and restrictions on outdoor activities. These measures effectively curbed the transmission of COVID-19. In December 2022, China gradually relaxed its NPIs, leading to notable shifts in the prevalence and spectrum of respiratory pathogens in infants [4, 5].

However, limited studies have explored changes in respiratory pathogens among infants under 3 months of age, a group characterized by minimal outdoor exposure, inconsistent mask use, and difficulties in maintaining hand hygiene across the pre-, peri-, and post-COVID-19 pandemic periods. To address this gap, this study retrospectively analyzed respiratory tract infections among hospitalized infants in Beijing from January 2018 to December 2023. Changes in the pathogen spectrum in infants before, during, and after the implementation of NPIs during the COVID-19 pandemic were analyzed. Our findings can provide clinical insights for improving the prevention and control of acute respiratory tract infections in local infants.

## Methods

### Inclusion criteria

Infants aged 0–3 months hospitalized with acute respiratory infections at the Children’s Hospital affiliated with the Capital Institute of Pediatrics between January 1, 2018, and December 31, 2023, were included in the study. These infants exhibited fever (body temperature ≥ 37.3 ℃) or respiratory symptoms (cough, sputum, difficulty breathing, or shortness of breath) and developed digestive symptoms such as poor appetite and vomiting after contact with individuals with respiratory infections. Additionally, all infants underwent testing for 14 respiratory pathogens.

### Exclusion criteria

Infants with congenital immunodeficiency or congenital genetic metabolic diseases were excluded. Those who developed respiratory infections during hospitalization were also excluded.

### Grouping

The COVID-19 epidemic began at the end of 2019, prompting the implementation of strict NPIs in Beijing starting in January 2020. These measures remained in place until the end of December 2022. Data were classified into three groups: the pre-epidemic (January 1, 2018, to December 31, 2019), epidemic prevention and control (January 1, 2020, to December 31, 2022), and post-epidemic (January 1, 2023, to December 31, 2023) groups. Infants were further stratified by age: 0–28 days, 29–60 days, and > 60 days.

### Specimen collection

Specimens, including throat swabs, sputum, or bronchoalveolar lavage fluid, were collected within 24 h of admission for respiratory pathogen nucleic acid testing. Thirteen multiplex detection kits (polymerase chain reaction capillary electrophoresis fragment analysis method) (Ningbo Haier Shi Gene Technology Co., Ltd., Ningbo, China; National Machinery Injection Standard 20,183,400,518) and pertussis bacteria nucleic acid detection kits (Shenzhen Yicubic Biotechnology Co., Ltd., Shenzhen, China; National Machinery Injection Standard 20,203,400,152) were used to detect respiratory pathogens. These included influenza A virus (Flu A), influenza B virus (Flu B), respiratory syncytial virus (RSV), parainfluenza virus (PIV), adenovirus (ADV), human metapneumovirus (HMPV), human bocavirus (HBoV), rhinovirus (HRV), coronavirus (CoV), *Chlamydia trachomatis* (Ct), *Chlamydia pneumoniae* (C.pn), *Mycoplasma pneumoniae* (MP), and *Bordetella pertussis* (BP). Beginning in January 2020, severe acute respiratory syndrome coronavirus 2 (SARS-CoV-2) was detected using novel coronavirus 2019-nCoV nucleic acid detection kits (Shanghai Bojie Medical Technology Co., Ltd., Shanghai, China; National Machinery Injection Standard 20,203,400,065). This study was approved by the Ethics Committee of the Capital Institute of Pediatrics, and written informed consent was not required (batch number: SHERLLM2024026).

### Statistical analysis

Statistical analyses were performed using SPSS 20.0 statistical software (IBM Corp., Armonk, NY, USA). Econometric data conforming to a normal distribution were expressed as mean ± standard deviation ($\overline{x}$ ± *s*), and intergroup comparisons were conducted using an independent sample t-test. Non-normally distributed data were represented by median (interquartile range: *M* (*P*_25_, *P*_75_), and intergroup comparisons were analyzed using the Wilcoxon rank-sum or Kruskal–Wallis test. Within-group comparisons of unified indicators were performed using one-way analysis of variance (ANOVA). Count data were expressed as numbers (percentages), with intergroup comparisons analyzed using chi-square tests and Fisher’s exact probability tests. Repeated measurement data were compared between groups using repeated measurement ANOVA. A *P*-value of less than 0.05 indicated statistically significant differences.

## Results

### General characteristics

From January 1, 2018, to December 31, 2023, a total of 8,743 infants were hospitalized, of whom 1,191 (13.6%) had acute respiratory infections. Seven infants were excluded owing to specific conditions: two with severe bronchopulmonary dysplasia, two with congenital developmental abnormalities, one with severe congenital heart disease, and two with hospital-acquired respiratory infections. Consequently, 1,184 infants were included in the study, comprising 649 men and 535 women. The mean age was 35.0 ± 20.85 days. Throat swab specimens were collected from all 1,184 infants, and 613 (51.8%) tested positive for at least one respiratory pathogen. The annual proportions of infants with acute respiratory infections from 2018 to 2023 were as follows: 15.0% (257/1708), 9.1% (174/1903), 12.0% (127/1057), 13.6% (165/1216), 11.7% (142/1213), and 19.4% (319/1646), respectively. The positive detection rates for respiratory pathogens during the same period were: 36.6% (94/257), 37.9% (66/174), 41.7% (53/127), 62.4% (103/165), 55.6% (79/142), and 68.3% (218/319), respectively. Moreover, the proportions of mixed infections caused by respiratory pathogens increased progressively: 3.2% (3/94), 7.6% (5/66), 7.5% (4/53), 12.6% (13/103), 11.4% (9/79), and 16.1% (35/218), respectively. From 2018 to 2023, the diversity of respiratory pathogens detected was 9, 6, 11, 7, 9, and 14 pathogens, respectively. Among the cases of mixed infections, 65 infants were co-infected with two pathogens, three were co-infected with three pathogens, and one was infected with four pathogens.

**Fig. 1:**
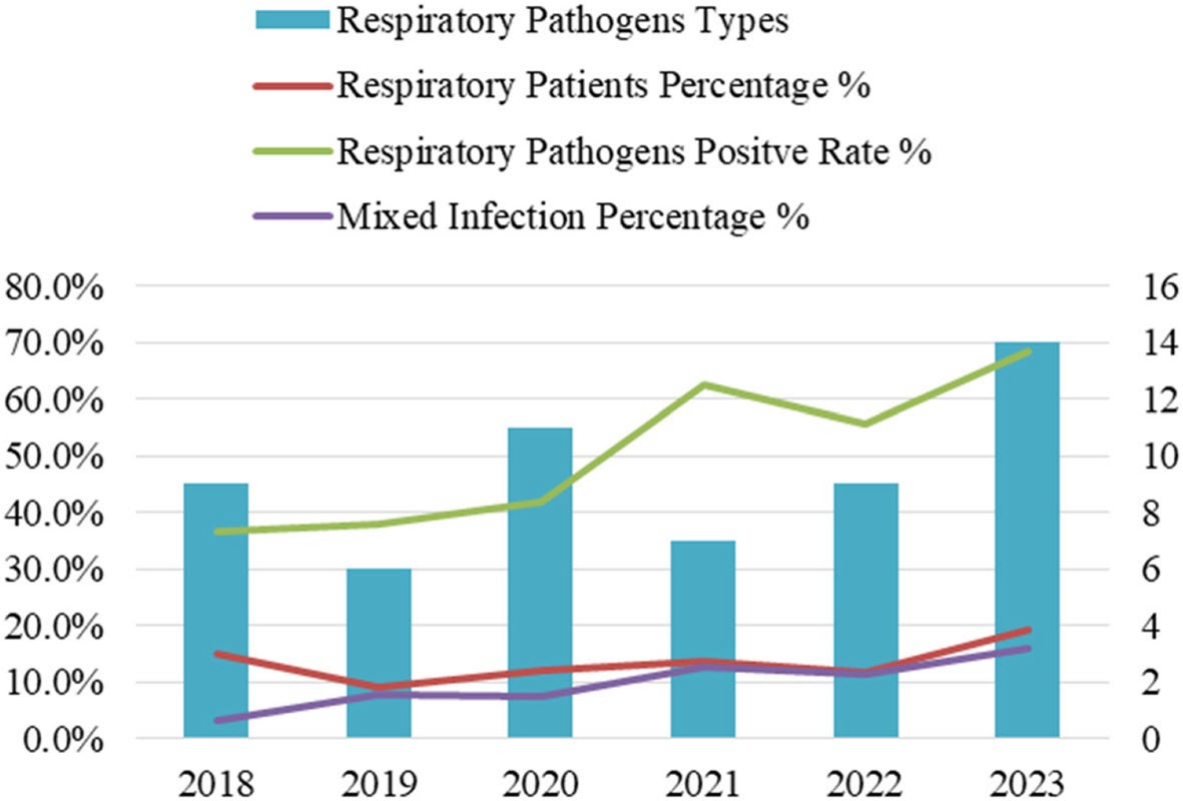
Proportion of infants with respiratory infections, positive detection rates of respiratory pathogens, mixed infection detection rates, and trends in respiratory pathogen types between 2018 and 2023

During the epidemic, the number of hospitalized infants significantly decreased. However, the proportion of infants with respiratory infections, positive detection rates of respiratory pathogens, mixed infection detection rates, and pathogen diversity gradually increased (Fig. 1). With detection rates of 28.5% (338/613), 5.9% (70/613), and 5.8% (69/613), the three most frequently detected pathogens were RSV, SARS-CoV-2, and PIV, respectively.

### Comparison of positive respiratory pathogens in infants before, during, and after the implementation of NPIs

From January 2018 to December 2019 (pre-epidemic), 3,611 infant patients were admitted, of whom 11.9% (431/3611) were diagnosed with acute respiratory infections. The positive detection rate of respiratory pathogens was 37.1% (160/431), and the mixed infection detection rate was 5.0% (8/160). During the epidemic (January 2020 to December 2022), 3,486 infants were admitted, of whom 12.4% (434/3486) were diagnosed with acute respiratory infections. The positive detection rate increased to 54.1% (235/434), and the mixed infection detection rate rose to 11.1% (26/235). In the post-epidemic period (January to December 2023), 1,646 infants were admitted, of whom 19.4% (319/1646) were diagnosed with acute respiratory infections. The positive detection rate reached 68.3% (218/319), and the mixed infection detection rate increased further to 16.1% (35/218).

The proportion of infants with respiratory infections, positive detection rates, and mixed infection detection rates significantly increased after the 2023 epidemic compared to before and during the epidemic levels (*χ*^*2*^ = 59.428, 73.088, 34.468, respectively; *P* < 0.05). Additionally, during the epidemic, detection rates of respiratory and mixed infections were significantly higher than those before the epidemic (*P* < 0.05).

Among infants testing positive for respiratory pathogens, no statistically significant differences (*P* > 0.05) were observed in sex, delivery method, proportion of full-term infants, or mechanical ventilation requirement across the three periods. However, significant differences (*P* < 0.05) were noted in infants testing positive for respiratory pathogens in the 0–28 days, 29–60 days, and > 60 days groups. Specifically, the proportion of newborns decreased, while the positive detection rates for infants aged 29–90 days increased significantly during and after the epidemic (*P* < 0.05). The admission weight and contact history with patients testing positive for respiratory pathogens significantly increased in the post-epidemic period compared to those before and during the epidemic (*P* < 0.05). The duration of pre-admission illness was shorter post-epidemic than during the epidemic (*P* < 0.05) but did not significantly differ from pre-epidemic levels (*P* > 0.05). Hospitalization duration was longer during and after the epidemic than before, with statistically significant differences (*P* < 0.05; Table 1).

**Table 1:** General information on infants testing positive for respiratory pathogens before, during, and after NPI implementation

	Before NPI implementation (160)	During NPI implementation (235)	After NPI release (218)	*χ*^*2*^	*P* Value
Gender				1.298	0.523
Male	93 (58.1)	136 (57.9)	116 (53.2)		
Female	67 (41.9)	99 (42.1)	102 (46.8)		
Age					
≤ 28d	93 (58.1)	91 (38.7)	68 (31.2)a	28.543	0.000
29 ~ 60d	55 (34.4)	124 (52.8)a	107 (49.1)a	13.737	0.001
> 60d	12 (7.5)	20 (8.5)	43 (19.7)ab	17.765	0.000
Full-term or premature infants				5.509	0.064
Term Infant	153 (95.6)	212 (90.2)	194 (89.0)		
Premature	7 (4.4)	23 (9.8)	24 (11.0)		
Respiratory patient contact history	78 (48.8)	114 (48.5)	150 (68.8)ab	23.241	0.000
Pre-admission course	3 (2, 5)	4 (2, 6)	3 (1, 5)b	H = 6.659	0.036
Hospitalization days	6 (4, 7)	7 (5, 9)a	6 (5, 8)a	H = 13.328	0.001
Mechanical ventilation use	15 (9.4)	27(11.5)	14 (6.4)	3.513	0.173

The difference between a and b was statistically significant compared to before and during the epidemic

*NPI *Non-Pharmaceutical Interventions

### Annual distribution of 14 respiratory pathogens

Before the COVID-19 outbreak (2018–2019), RSV accounted for 30.5% (53/174) of pathogens in hospitalized infants with acute respiratory infections, followed by PIV (5.1%, 13/257) and MP (4%, 7/174). After strict NPIs were implemented in Beijing in late January 2020, the annual detection rate of RSV in 2020 reduced to 18.1% (23/127). Conversely, the annual detection rates of HRV and C.pn increased to 10.3% (17/127) and 9.7% (16/127), respectively, from pre-COVID-19 rates of 0.6% (1/174) and 1.2% (3/257). BP was not detected in 2020 (0%). In 2021, RSV (38.2%, 63/165), HRV (10.3%, 17/165), and C.pn (9.7%, 16/165) remained the top three pathogens detected. Following the relaxation of prevention measures in December 2022, the detection rates of Flu B (4.2%, 6/142), MP (7.0%, 10/142), and BP (0.7%, 1/142) increased slightly, while other respiratory pathogens exhibited a downward trend.

**Fig. 2:**
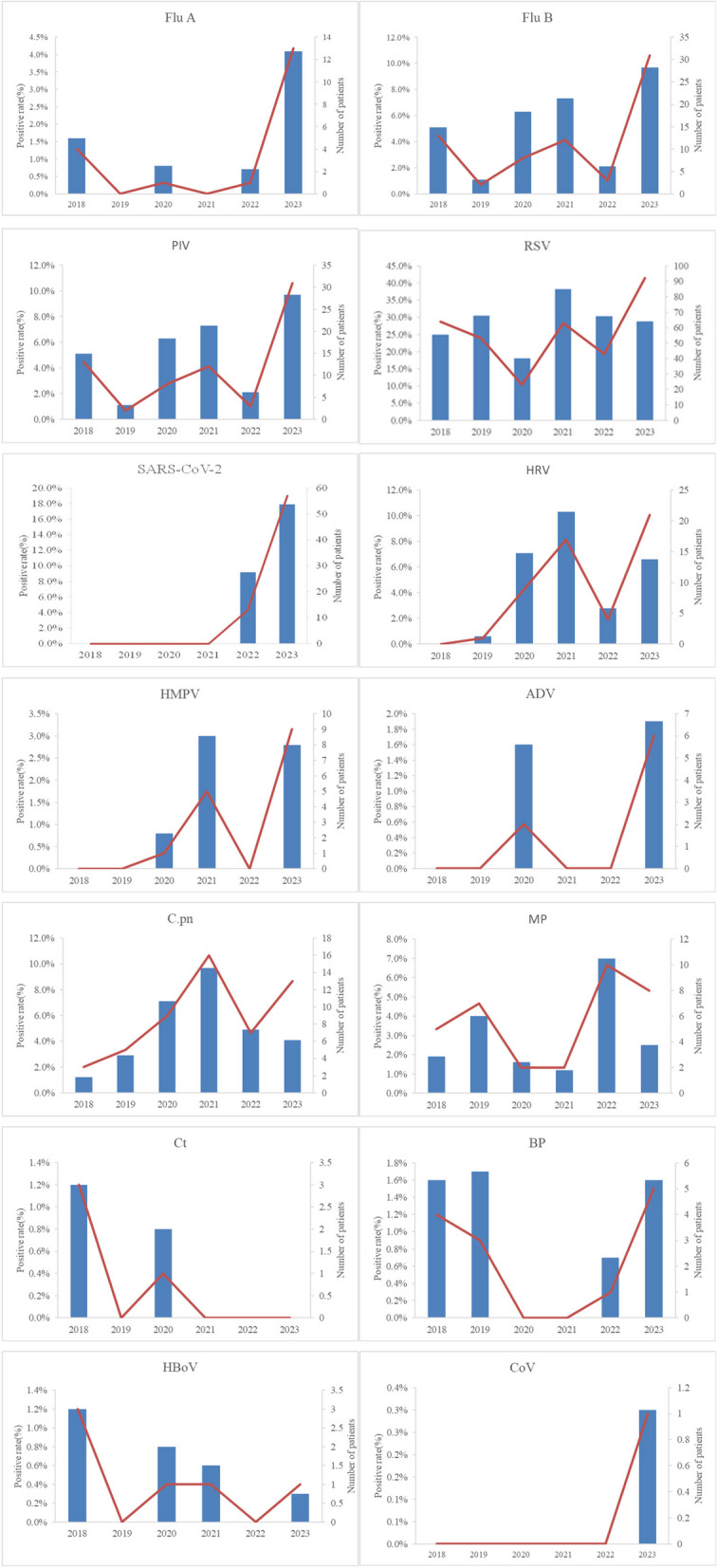
Detection rates and counts of positive cases for 14 respiratory pathogens among hospitalized infants in Beijing from January 2018 to December 2023. ADV - Adenovirus; BP - *Bordetella pertussis*; C.pn - *Chlamydophila pneumoniae*; CoV - Coronavirus; Ct - *Chlamydia trachomatis*; hbOv - Human Bocavirus; HMPV - Human Metapneumovirus; HRV - Human Rhinovirus; MP - *Mycoplasma pneumoniae*; PIV - Parainfluenza Virus; RSV - Respiratory Syncytial Virus; SARS-CoV-2 - Severe Acute Respiratory Syndrome Coronavirus 2

After the lifting of NPIs in 2023, the positive detection rate of respiratory pathogens significantly increased to 68.3% (218/319), with all pathogens being detected. Thirteen respiratory pathogens were detected throughout the year. Notably, the numbers of positive cases of Flu A, PIV, RSV, SARS-CoV-2, HRV, HMPV, ADV, and BP significantly increased compared to the pre-epidemic and epidemic prevention and control periods. The top three pathogens identified were RSV (28.8%, 92/319), SARS-COV-2 (17.9%, 57/319), and PIV (9.7%, 31/319). Detailed data are presented in Table 2 and Fig. 2.

**Table 2:** Annual distribution of 14 respiratory pathogens (%)

	Number of respiratory tract infections (*n* = 1184)	2018 (*n* = 257)	2019 (*n* = 174)	2020 (*n* = 127)	2021 (*n* = 165)	2022 (*n* = 142)	2023 (*n* = 319)
Respiratory pathogen positive	613 (51.8)	94 (36.6)	66 (37.9)	53 (41.7)	103 (62.4)	79 (55.6)	218 (68.3)
mixed infection	69 (5.8)	3 (1.2)	5 (2.9)	4 (3.1)	13 (7.9)	9 (6.3)	35 (11.0)
Flu A	19 (1.6)	4 (1.6)	0	1 (0.8)	0	1 (0.7)	13 (4.1)
Flu B	9 (0.8)	1 (0.4)	0	1 (0.8)	0	6 (4.2)	1 (0.3)
PIV	69 (5.8)	13 (5.1)	2 (1.1)	8 (6.3)	12 (7.3)	3 (2.1)	31 (9.7)
RSV	338 (28.5)	64 (24.9)	53 (30.5)	23 (18.1)	63 (38.2)	43 (30.3)	92 (28.8)
SARS-COV-2	70 (5.9)	0	0	0	0	13 (9.2)	57 (17.9)
HRV	52 (4.4)	0	1 (0.6)	9 (7.1)	17 (10.3)	4 (2.8)	21 (6.6)
HMPV	15 (1.3)	0	0	1 (0.8)	5 (3.0)	0	9 (2.8)
ADV	8 (0.7)	0	0	2 (1.6)	0	0	6 (1.9)
C.pn	53 (4.5)	3 (1.2)	5 (2.9)	9 (7.1)	16 (9.7)	7 (4.9)	13 (4.1)
MP	34 (2.9)	5 (1.9)	7 (4.0)	2 (1.6)	2 (1.2)	10 (7.0)	8 (2.5)
Ct	4 (0.3)	3 (1.2)	0	1 (0.8)	0	0	0
BP	13 (1.1)	4 (1.6)	3 (1.7)	0	0	1 (0.7)	5 (1.6)
HBoV	6 (0.5)	3 (1.2)	0	1 (1.9)	1 (0.6)	0	1 (0.3)
CoV	1 (0.1)	0	0	0	0	0	1 (0.3)

*ADV* Adenovirus, *BP Bordetella pertussis*, *C.pn Chlamydophila pneumoniae*, *Ct Chlamydia trachomatis*, *Flu A* Influenza A virus, *Flu B* Influenza B virus, *HBoV* Human Bocavirus, *HMPV* Human Metapneumovirus, *HRV* Human Rhinovirus, *MP Mycoplasma pneumoniae*, *n* Number, *PIV* Parainfluenza Virus, *RSV* Respiratory Syncytial Virus, *SARS-COV-2* Severe Acute Respiratory Syndrome Coronavirus 2

### Monthly and quarterly distributions of 14 respiratory pathogens

**Fig. 3:**
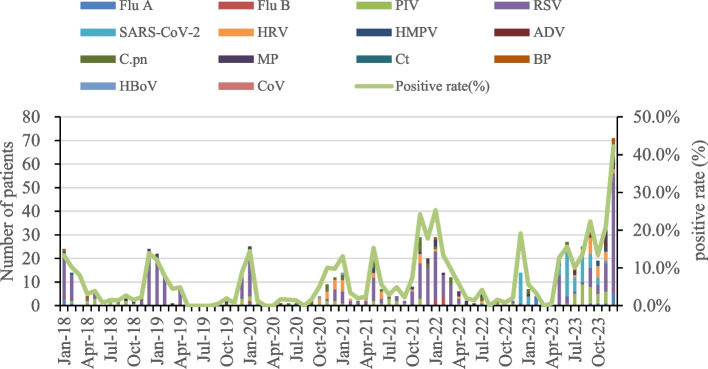
Monthly distribution and hospitalization proportions of hospitalized infants testing positive for 14 respiratory pathogens from 2018 to 2023. ADV - Adenovirus; BP - *Bordetella pertussis*; C.pn - *Chlamydophila pneumoniae*; CoV - Coronavirus; Ct - *Chlamydia trachomatis*; Flu A - Influenza A virus; Flu B - Influenza B virus; HBoV - Human Bocavirus; HMPV - Human Metapneumovirus; HRV - Human Rhinovirus; MP - *Mycoplasma pneumoniae*; PIV - Parainfluenza Virus; RSV - Respiratory Syncytial Virus; SARS-CoV-2 - Severe Acute Respiratory Syndrome Coronavirus 2

**Fig. 4:**
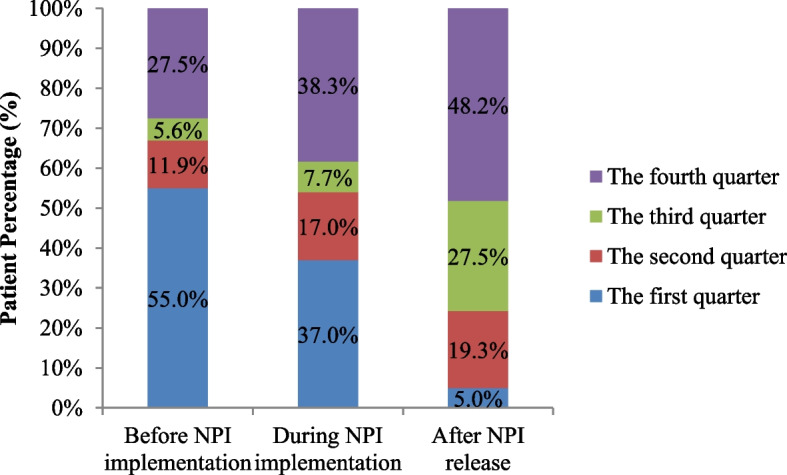
Quarterly distribution of respiratory pathogens before, during, and after the epidemic. NPI - Non-Pharmacological Interventions

Before the epidemic, the incidence of acute respiratory infections among infants exhibited a distinct seasonal “V” pattern, with peaks occurring between November to February, as illustrated in Fig. 3. Quarterly distributions exhibited higher proportions of cases in the first and fourth quarters (Fig. 4).

With the implementation of NPIs in Beijing at the end of January 2020, the proportion of hospitalized infants testing positive for respiratory pathogens reduced significantly from 14.4% (22/153) in January to 1.3% (1/76) in February. By December 2020, only 9.8% (11/112) of hospitalized infants tested positive, including two cases of PIV, four of RSV, four of HRV, and one of RSV and C.pn coinfection (Fig. 3). The proportions of infants with respiratory infections in the first and fourth quarters of 2020 were lower than the pre-epidemic levels.

In January 2021, the proportion of respiratory-pathogen-positive hospitalizations was 13.1% (11/84), almost returning to pre-pandemic levels. A notable peak occurred in May 2021, with 15.3% (19/124) of cases primarily owing to RSV infection. From October 2021 to March 2022, respiratory infections, primarily caused by RSV, peaked at 25.3% (25/99), exceeding pre-pandemic levels. The detection rates of respiratory pathogens in October and November of the fourth quarter were at a relatively low level. However, in December 2022, a new peak dominated by SARS-CoV-2 infections emerged (13 cases with a minimum age of 1 day, and 9 patients aged ≤ 7 days), and 19.1% (13/68) patients were hospitalized (Fig. 3). Proportions of respiratory pathogens in the second and fourth quarters during the epidemic significantly exceeded pre-epidemic levels (*P* < 0.05; Table 3 and Fig. 4).

**Table 3:** Quarterly comparison of respiratory pathogens before, during, and after the epidemic

	Before NPI implementation (431)	During NPI implementation (434)	After NPI release (319)	*χ*^*2*^ Value	*P* Value
The first quarter	88 (20.4)	87(20.0)	11(3.4)ab	12.336	0.002
The second quarter	19(4.4)	40(9.2)a	42(13.2)a	24.617	0.000
The third quarter	9(2.1)	18(4.1)	60(18.8)ab	45.785	0.000
The fourth quarter	44(10.2)	90(20.7)a	105(32.9)ab	35.651	0.000

Statistical difference between a and b was observed during the epidemic compared to before and after the epidemic. NPI—Non-Pharmaceutical Interventions

From January to April 2023, the detection rates of respiratory pathogens remained very low. From May of the second quarter, the number of positive cases of respiratory pathogens reached 12.8% (19/149), primarily owing to RSV (16/19). In June, the proportion of positive cases of respiratory pathogens reached 15.6% (22/141), primarily owing to SARS-COV-2 (21/22). By December, the proportion of hospitalized infants testing positive for respiratory pathogens reached a new peak of 42.2% (62/147) after the epidemic, primarily driven by RSV infections (49 cases). The number of RSV cases co-infected with BP, C.pn, MP, and ADV also increased compared to levels observed before and during the epidemic. Notably, no SARS-CoV-2 cases were detected between February and April or December after the epidemic, while cases were identified in all other months (Fig. 3). Compared to before and during the pandemic, the proportions and detection rates of respiratory pathogens after the epidemic were lower in the first quarter but higher in the third and fourth quarters, with a statistically significant difference (*P* < 0.05; Table 3 and Fig. 4).

### Changes in respiratory pathogens before, during, and after the epidemic

Statistically significant differences were observed in the detection rates of Flu A, PIV, SARS-CoV-2, HRV, HMPV, ADV, and C.pn before, during, and after the implementation of NPIs during the COVID-19 pandemic (*P* < 0.05). After the epidemic, the detection rates of Flu A, PIV, and SARS-CoV-2 were significantly higher than those before and during the epidemic (*P* < 0.017). Similarly, the detection rates of HRV, HMPV, and ADV increased significantly after the epidemic compared to before (*P* < 0.017), although their rates during the epidemic remained relatively unchanged (*P* > 0.017).

**Fig. 5:**
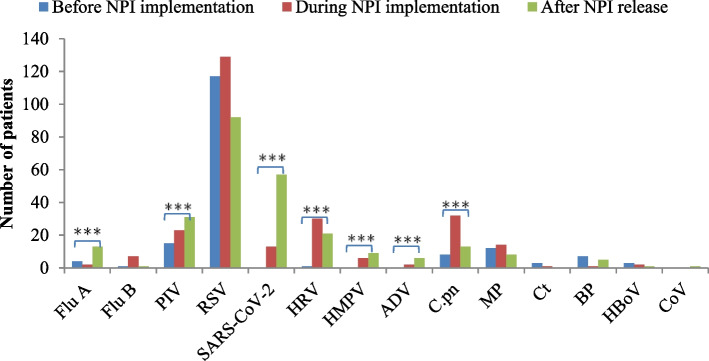
Comparison of the detection rates of 14 positive respiratory pathogens in hospitalized infants in Beijing before, during, and after the epidemic. ADV—Adenovirus; BP—*Bordetella pertussis*; C.pn—*Chlamydophila pneumoniae*; CoV—Coronavirus; Ct—*Chlamydia trachomatis*; Flu A—Influenza A virus; Flu B—Influenza B virus; HBoV—Human Bocavirus; HMPV—Human Metapneumovirus; HRV—Human Rhinovirus; MP—*Mycoplasma pneumoniae*; NPI—Non-Pharmacological Interventions; PIV—Parainfluenza Virus; RSV—Respiratory Syncytial Virus; SARS-CoV-2—Severe Acute Respiratory Syndrome Coronavirus 2

The detection rate of C.pn increased significantly during the epidemic compared to the pre-epidemic period (*P* < 0.017), but no statistically significant change was observed after the epidemic (*P* > 0.017). In contrast, the detection rates of Flu B, RSV, MP, Ct, BP, HBoV, and CoV exhibited no significant changes before, during, and after the epidemic, as detailed in Table 4 and Fig. 5.

**Table 4:** Changes in respiratory pathogens before, during, and after the epidemic

	Before NPI implementation (431)	During NPI implementation (434)	After NPI release (319)	*χ*^*2*^ Value	*P* Value
Flu A	4 (0.9)	2 (0.5)	13 (4.1)ab	17.177	0.000
Flu B	1 (0.2)	7 (1.6)	1 (0.3)	5.448	0.065*
PIV	15 (3.5)	23 (5.3)	31 (9.7)ab	13.345	0.001
RSV	117 (27.1)	129 (29.7)	92 (28.8)	0.723	0.697
SARS-COV-2	0	13 (3.0)a	57 (17.9)ab	115.698	0.000
HRV	1 (0.2)	30 (6.9)a	21 (6.6)a	27.976	0.000
HMPV	0	6 (1.4)a	9 (2.8)a	11.739	0.003
ADV	0	2 (0.5)	6 (1.9)a	8.644	0.004*
C.pn	8 (1.9)	32 (7.4)a	13 (4.1)	15.558	0.000
MP	12 (2.8)	14 (3.2)	6 (1.9)	11.282	0.527
Ct	3 (0.7)	1 (0.2)	0	2.186	0.389*
BP	7 (1.6)	1 (0.2)	5 (1.6)	5.361	0.073*
HBoV	3 (0.7)	2 (0.5)	1 (0.3)	0.582	0.777*
CoV	0	0	1 (0.3)	2.274	0.269*

^*^Fisher’s exact probability test: Statistically significant difference between a and b before and during the pandemic is observed

*ADV* Adenovirus, *BP* Bordetella pertussis, *C.pn Chlamydophila pneumoniae*, *CoV* Coronavirus, *Ct Chlamydia trachomatis*, *Flu A* Influenza A virus, *Flu B* Influenza B virus, *HBoV* Human Bocavirus, *HMPV* Human Metapneumovirus, *HRV* Human Rhinovirus, *MP Mycoplasma pneumoniae*, *NPI* Non-Pharmaceutical Interventions, *PIV* Parainfluenza Virus, *RSV* Respiratory Syncytial Virus, *SARS-COV-2* Severe Acute Respiratory Syndrome Coronavirus 2, χ2 Chi-squared

## Discussion

Various respiratory viruses, as well as MP, C.pn, and other pathogens, are the main causes of respiratory infections in infants. Acute respiratory infections are a common cause of outpatient visits and hospitalization in this population. Both before and after the COVID-19 pandemic, respiratory diseases in infants worldwide posed a substantial burden on global healthcare systems, with hospitalizations accounting for 19.3% of cases. Among infants with RSV infections, 59% were under 6 months old, and those under 2 months were at a higher risk of hospitalization [6, 7]. This study retrospectively analyzed the detection rates and trends of respiratory pathogens in infants aged under 3 months hospitalized in Beijing before, during, and after the outbreak of COVID-19. The overall positive detection rate of respiratory pathogens was 51.8%, consistent with the previously reported rate of 48.4% [8].

Following the global outbreak of COVID-19 in late 2019, the implementation of NPIs led to a marked reduction in respiratory infections among infants [8–10]. The results of this study revealed that the number of hospitalized infants with respiratory infections decreased because of the 2020 epidemic prevention and control measures. Still, the positive detection rate of pathogens did not decline. This observation aligns with a previous study on the detection rate of respiratory pathogens in preschool children in Henan province [8]. The implementation of strict NPIs against COVID-19 likely contributed to this outcome by effectively blocking the transmission of other respiratory viruses. The notion of minimizing hospital visits may have discouraged parents from seeking medical care for infants with mild symptoms; however, it did not deter parents from seeking treatments for infants with more severe symptoms. This likely contributed to the reduction in the absolute number of respiratory infections but not in the positive detection rate of respiratory pathogens.

During the COVID-19 pandemic, global adjustments to NPIs influenced the dynamics of respiratory infections. Following the relaxation of NPIs, respiratory pathogens rebounded, and co-infections among children became more prevalent [7, 9, 11]. This study revealed that while the number of hospitalized infants with respiratory infections during the epidemic prevention and control period was lower than that before the epidemic, the proportion of respiratory cases, positive detection rate of respiratory pathogens, and mixed infection rates have been increasing annually.

In 2023, after the relaxation of NPIs, 319 infants with acute respiratory infections were hospitalized, accounting for 19.4% of hospitalizations. The positive detection rate of respiratory pathogens was 68.3% (218/319), and the mixed infection rate was 17.4% (38/218), both significantly higher than pre-epidemic and epidemic levels. The detection of respiratory pathogens peaked, with 13 species identified throughout the year and 10 species detected in November alone. Following the release of NPIs, the number of infants hospitalized with acute respiratory infections sharply increased, reaching the highest levels recorded since 2018. Similarly, the incidence of mixed infections increased significantly. These trends may stem from reduced maternal immunity to respiratory pathogens during the epidemic prevention and control period, potentially affecting infants’ immune function at birth. Furthermore, the increase in birth rates during this period and the complete lifting of NPIs likely contributed to outbreaks of respiratory infections among infants [12, 13]. The implementation of NPIs may have reduced immune stimulation, leaving the population more susceptible to respiratory infection and lowered overall immunity, creating an “immune gap” compared to pre-epidemic levels [14, 15]. The findings of this study align with previous reports, such as Bobby et al. [3], which link the release of NPIs to a resurgence in respiratory pathogens. To mitigate future peaks of respiratory infections, preventive and control measures, such as wearing masks and enhanced hand hygiene, should be considered to reduce respiratory infections in infants.

Ebba et al. [7] reported that infants under 1 year of age, particularly male infants younger than 2 months, are more likely to be hospitalized for respiratory diseases. However, our findings indicate no statistically significant differences in the sex distribution of infants with positive respiratory pathogen tests before, during, and after the COVID-19 pandemic. Interestingly, the proportion of newborns among infants with positive respiratory pathogens decreased post-pandemic. In contrast, the number of older infants (aged 29–90 days) increased significantly during and after the epidemic prevention and control period. This may be because, following the COVID-19 outbreak, newborns experienced minimal exposure to the external environment, with strict adherence to hand hygiene by caregivers and protective isolation measures against respiratory infections. Notably, older infants tend to go out more frequently than newborns, likely due to the gradual weakening of protective isolation practices. Additionally, the higher levels of maternal antibodies in the newborn may reduce their risk of respiratory infection.

Droplets and aerosols are common routes of respiratory virus transmission, with direct contact being particularly significant for young infants. The findings of this study corroborate this, revealing that 68.8% of infants testing positive for respiratory pathogens after the epidemic had a contact history of respiratory infections. This rate was significantly higher than that observed before and during the epidemic. During the long-term implementation of prevention and control measures, the diversity and likelihood of pathogen exposure in infants were reduced, resulting in reduced immune establishment and maturation due to limited pathogen stimulation [15]. Following the relaxation of these measures, infants were more frequently exposed to a broader range of pathogens, resulting in more complex clinical manifestations, especially in immunocompromised children. This increased exposure likely shortened the time from symptom onset to medical care. Additionally, the post-pandemic hospitalization duration for infants was significantly during the pre-pandemic and pandemic periods. Notably, there was no increase in the incidence of respiratory tract infections requiring mechanical ventilation in critically ill infants across the three time periods, consistent with the findings reported by Naishisha [16]. Mixed infections also did not elevate the proportion of critically ill infants. The extended hospitalization time may be attributed to the higher frequency of pathogen infection and prolonged symptom duration observed after the epidemic.

The outbreak of COVID-19 and the implementation and subsequent lifting of NPIs induced notable changes in respiratory pathogens in infants before and after the epidemic. RSV was the primary pathogen responsible for acute respiratory infections and hospitalization in infants [16–19]. This study confirms that RSV was the leading cause of acute respiratory infections in infants hospitalized aged 0–3 months before the epidemic. However, during the pandemic, NPIs altered the proportions of respiratory viruses detected in preschool children and reduced the infection rates of enveloped viruses [3, 5, 20–22] while leaving those of non-enveloped viruses unaffected [8, 23–26]. Specifically, the RSV-positive detection rate in 2020 was only 18.1%, significantly lower than the pre-epidemic range of 24.9%–30.5%. In contrast, non-enveloped viruses such as HRV, ADV, and C.pn exhibited increasing trends. This pattern may be attributed to the unique characteristics of nonenveloped viruses, including superior thermal stability, tolerance to dry and acidic environments, and lower sensitivity to alcohol [27]. These features enable non-enveloped viruses to persist longer in external environments, maintaining their activity and infectivity under adverse conditions. Furthermore, they possess advantages in transmission via aerosols and direct contact [28–30].

Robert et al. [14] reported that prolonged low-dose or absent exposure to pathogens may weaken immunity and increase susceptibility within the population. Consequently, the relaxation of NPIs may leave a significant portion of the population more vulnerable to respiratory pathogens. This study found that the detection rates of RSV and HMPV returned to pre-pandemic levels in 2021, while PIV, HRV, and C.pn maintained high detection rates from 2020 to 2021. Notably, BP was not detected in either 2020 or 2021. In 2022, the relaxation of NPIs coincided with the emergence of SARS-COV-2 in newborns and infants. The detection rates of RSV, PIV, HRV, and C.pn slightly declined, while ADV and HMPV were not detected throughout the year. However, MP and Flu B exhibited an increasing trend, and BP was detected again. Parsa et al. [31] similarly observed an increase in non-COVID-19 respiratory pathogens following NPI relaxation.

This study also demonstrated that the detection rates of Flu A, PIV, HRV, HMPV, and ADV in hospitalized infants significantly exceeded pre-epidemic levels after the release of NPIs. Additionally, SARS-CoV-2 emerged as a major cause of hospitalization in infants aged 0–3 months. During the epidemic prevention and control period, the detection rate of C.pn increased compared to pre-epidemic levels but stabilized after the epidemic. The detection rates of Flu B, RSV, MP, Ct, BP, HBoV, and CoV did not show significant variation across the three epidemic phases. However, after the release of NPIs, the number of mixed infections in infants increased significantly. From November and December 2023, 9–10 species of respiratory pathogens were detected monthly, and the mixed infection rate of RSV with BP, C.pn, MP, and ADV reached 16.1%, over three times higher than pre-epidemic levels. The number of detected cases of Flu A, PIV, RSV, SARS-CoV-2, HRV, HMPV, ADV, and BP in hospitalized infants reached its highest level in 6 years. In the future, the lifting of epidemic-related NPIs could potentially lead to a further increase in respiratory pathogens. Therefore, it is essential to remain vigilant, ensuring close monitoring and the implementation of effective measures for the prevention and management of these respiratory pathogens.

After the COVID-19 outbreak and the implementation of NPIs, the seasonality of respiratory pathogens in infants was disrupted, leading to changes in infections with seasonal respiratory pathogens such as RSV. This study showed that before the COVID-19 epidemic, the epidemic season of RSV infection in hospitalized infants aged 0–3 months in Beijing began in November or December and lasted until February, spanning 3–4 months. This observation is consistent with previous reports by Movva et al. [16], Li et al. [32], and Bardsley et al. [33]. However, the strict NPIs implemented at the end of January 2020 effectively ended the RSV epidemic.

The subsequent adjustment and lifting of NPIs may have led to the resurgence of seasonal viruses such as PIV and RSV, with shifts in their seasonal patterns [31]. This study observed no peak in RSV prevalence during the winter of 2020–2021 compared to pre-pandemic levels, along with a decreased positive detection rate of respiratory pathogens in the first quarter. However, an RSV outbreak occurred in May 2021, aligning with previously reported peak incidences in infants [16, 33–37]. Another significant peak occurred in October 2021, with a detection rate of (25.3%), predominantly driven by RSV. Simultaneously, pathogens, including HRV, HMPV, C.pn, and MP, contributed to infections lasting over 5 months. The positive hospitalization rate of respiratory pathogens exceeded pre-epidemic levels. These changes may be due to the relaxation of epidemic prevention and control measures, reduced reduction in caregiver vigilance regarding infant protection, and increased risk of cross-infection as primary and secondary schools reopened in September 2021. Another contributing factor could be the lack of natural immunity to RSV among infants born during the winter of 2021, when RSV activity was minimal. Delayed or missed vaccinations due to prevention and control measures may have further lowered population immunity. Starting in December 2022, NPIs were gradually lifted, coinciding with a surge in SARS-CoV-2 infections. By May 2023, an increase in RSV prevalence emerged, followed by another peak of SARS-CoV-2 infection in June. This contributed to an elevated positive detection rate of respiratory pathogens. This trend indicates off-season outbreaks of respiratory pathogens during the second quarter. The proportion of RSV infections remained relatively stable over the years except for 2020 the absolute number of admissions has increased, particularly from October 2023 onward. By December, RSV-related respiratory infections reached the highest level in 6 years, and the hospitalization of pathogen-positive infants reached a new peak of 42.2% (62/147), a threefold increase from pre-epidemic levels.

One year after lifting of the NPIs, the respiratory pathogen epidemic extended to 8 months. As reported by Rachel et al. [5], the lifting of the NPIs, the increase in susceptible populations, and the overlapping of respiratory pathogen epidemic seasons may explain the sharp rise in the number of patients with respiratory infections. In the post-NPI era, the seasonal outbreak of respiratory pathogens in young infants may gradually return to pre-COVID-19 levels. However, ongoing surveillance and preventive measures are essential to mitigate the risk of respiratory infections in this vulnerable population.

Our study has some limitations. First, its retrospective, single-center design may limit the generalizability of the findings. Second, before and during the epidemic prevention and control period, some pathogens, such as HMPV and HRV, were not consistently monitored, which may have influenced the results.

## Conclusions

This study analyzed changes in respiratory pathogens among infants under 3 months of age from 2018 and 2023. The COVID-19 outbreak and subsequent lifting of NPIs led to an increase in hospitalization rate, the positive detection rate of respiratory pathogens, the proportion of mixed infections, and the diversity of detected pathogens in infants with respiratory infections. RSV remained the primary pathogen in hospitalized infants, but notable increases in SARS-CoV-2, PIV, and HRV were observed compared to pre-epidemic levels. Within 1 year of NPI relaxation, off-season epidemics of respiratory pathogens occurred among infants under 3 months of age. Special attention should be directed towards preventing respiratory infections in infants under 3 months of age during periods of heightened respiratory pathogen activity.

## Data Availability

Further information and requests for resources should be directed to and will be fulfilled by the lead contact, Yajuan Wang. The data reported in this paper may be shared upon request, subject to approval by the relevant ethics and regulatory committees. This study did not report the original code. Any additional information required to reanalyze the data reported in this paper is also available from the lead contact upon request.

## Abbreviations

NPIs: Non-pharmacological interventions
Flu A: Influenza virus A
Flu B: Influenza virus B
RSV: Respiratory syncytial virus
PIV: Parainfluenza virus
ADV: Adenovirus
HMPV: Human metapneumovirus
HBoV: Human bocavirus
HRV: Human rhinovirus
CoV: Coronavirus
Ct: *Chlamydia trachomatis*
C.pn: *Chlamydia pneumoniae*
MP: *Mycoplasma pneumoniae*
BP: *Bordetella pertussis*
SARS-CoV-2: Severe acute respiratory syndrome coronavirus 2
